# A pilot study comparing the prevalence of orthorexia nervosa in regular students and those in University sports teams

**DOI:** 10.1007/s40519-018-0584-0

**Published:** 2018-09-27

**Authors:** Tom Clifford, Charlotte Blyth

**Affiliations:** 1 Institute of Cellular Medicine, Newcastle University, Newcastle-upon-Tyne, UK; 2 School of Biomedical Sciences, Newcastle University, Newcastle-upon-Tyne, UK; 3 Faculty of Medical Sciences, School of Biomedicine, Newcastle University, Newcastle-upon-Tyne, NE2 4HH UK

**Keywords:** Sport, Exercise, Eating disorders, Mental health, Orthorexia nervosa, ORTO-15, Students, Athletes

## Abstract

**Purpose:**

Orthorexia nervosa (ON) is a pattern of eating characterized by a pathological fixation on restricting foods based on their perceived health. Like many eating disorders, ON is thought to be more prevalent in athletes. This was a preliminary study to explore the prevalence of ON in University students to determine whether those who compete in University sports have higher orthorexic tendencies.

**Methods:**

116 male and female student athletes (age 21 ± 1 years) and 99 non-athlete controls (21 ± 2) from Universities in the North East of the UK completed the ORTO-15 test (≤ 40 being the cutoff) used to screen individuals for ON.

**Results:**

ON symptoms were high in all students (76%); there was no difference in ORTO-15 scores between the athletes (36.6 ± 3.9) and non-athlete controls (37.2 ± 3.8; *P* = 0.279). There was a difference in scores between those who completed ≥ 10 h of exercise per week (36.65 ± 4.38) and those who do ≤ 10 h a week (37.38 ± 3.65) (*P* = 0.008; ES = 0.43). ORTO-15 scores were not higher in athletes competing in aesthetic and weight dependent sports (*P* > 0.05).

**Conclusions:**

Being a student athlete for a University sports team did not affect ON prevalence; however, there appears to be a greater risk for students in general, and for athletes who undertake high volumes of exercise. Nonetheless, the high prevalence of ON symptoms may be attributed to flaws in the ORTO-15 questionnaire, and therefore, future studies should focus on developing a more valid method for ON diagnosis.

**Level of evidence:**

III, case-control study.

## Introduction

Orthorexia nervosa (ON) is a pattern of eating characterized by a pathological fixation on consuming foods based on their quality, rather than their quantity [1]. While it has many parallels to anorexia nervosa, a recognized eating disorder where affected individuals restrict food in the pursuit of becoming thin [2], orthorexic sufferers will restrict food due to what they perceive to be ‘impure’ or ‘unhealthy’, as their main motivation is to achieve optimal health and/or avoid illness, rather than weight loss [3]. However, as a result of extreme restriction or preoccupation with their diet, ON sufferers might eliminate entire food groups which can lead to nutritional deficiencies, malnourishment and weight loss [4, 5]. Orthorexic individuals may also suffer from mental health issues such as anxiety or depression [6], and may become socially isolated due to their rigid eating patterns [4, 5, 7].

Despite the growing interest in ON, it is still not recognized by the Diagnostic and Statistical Manual of Mental Disorders (DMV-5) as an eating disorder [7–9]. Nevertheless, there is an increased awareness amongst eating disorder specialists in the Netherlands, suggesting that amongst healthcare professionals, there may be a growing understanding and recognition of ON as an eating disorder and this may help with diagnosis and treatment in the future [10, 11].

In the past two decades, an increasing amount of attention has been given towards eating disorders in sport and exercise populations [12]. Although not consistent, the current consensus is that eating disorders are thought to be more common in athletes compared to the general public [13–15]. Indeed, a large study by Sundgot-Borgen et al. [13] found that symptoms of eating disorders were far higher in athletes compared to non-athletes (13.5 vs. 4.6%, respectively). In a more recent study, the risk of developing an eating disorder was ~ 5% higher in athletes than non-athlete controls based on clinical interviews [15].

Eating disorders are potentially very harmful to an athlete, affecting both performance and overall health. Indeed, athletes who have an eating disorder perform more inconsistently, are more vulnerable to injuries, and have shorter careers [16]. Furthermore, the mortality rate amongst those who have eating disorders is high [17] and for those who survive, many never fully recover and remain ill, mentally and/or physically, for the rest of their lives [18].

Because it is normal for an athlete to maintain control over their diet to enhance their performance, it may be harder to detect ON in the sporting population. As such, until recently, the prevalence of ON in athletes has been relatively unknown [19]. However, in the first study to report on the prevalence of orthorexia in athletes, Segura-Garcia et al. [19] found a high frequency of orthorexic tendencies in both male and female athletes (30 and 28%, respectively) across a wide range of sports. They also highlighted the difficulty for coaches and other sporting managers of being aware of and recognizing ON due to the symptoms often being masked by athletes’ general desire to eat better for performance.

Some recent studies have suggested that there is a link between ON and exercise habits both in students and the general population [20–22]. One of the studies in a student population found that orthorexic eating habits were associated with high exercise volumes suggesting students who play sports and, therefore, exercise more frequently, might display higher ON symptoms [21]. However, to date, no study has exclusively investigated the prevalence of ON symptoms in University student athletes. Thus, the primary aim of this study was to assess the prevalence of ON in students competing in the British University and College Sports (BUCS) while studying at University compared to students who were not. Teams competing in BUCS are known to be of a high standard, with over 60% of British Olympic gold medalists in the past 25 years having competed previously in a BUCS league [23]. This high standard would suggest that some athletes competing in these leagues may be at a greater risk of ON due to the pressure associated with more competitive leagues and competitions. We wish to make it clear that this was designed as a small-scale pilot study that aimed to develop our understanding of ON in student athletes and evaluate whether a larger scale study is warranted in this potentially high-risk population.

## Methods

### Participants

Ethical approval was sought and granted by Newcastle University Faculty of Medical Sciences Ethics Committee. Undergraduate and postgraduate students studying at Universities in the North East of the UK volunteered for this study. Students were recruited via direct contact with presidents or representatives of sports clubs, University societies and University courses, who then sent an email to their current members inviting them to participate. Inclusion criteria included being a current member of a University team; this was indicated on the survey by asking they current play a sport with a yes or no option available and in what sport they currently competed in at the University. The data was collected in November and December of 2017 and aimed to get a mixture of students who competed for the University sports teams, and those who do not—who acted as an age-matched control. Data was collected and analyzed for 215 students in total; 116 competed for a University team and were, therefore, considered student athletes, and the remaining 99 were not members of a University sports team (or any others), and used as non-athlete controls. Descriptive data for the participants in each group is displayed in Table 2. Both sets of students came from a diverse range of degree programmes that included but was not limited to Medicine, Dentistry, Food and Human Nutrition, Economics, Geography, Computer Science, Linguistics and Marine Biology.

### Survey

Participants were emailed a link to complete the online survey. This took approximately 10 min after first reading the description of the study and providing informed consent. The survey consisted of several sections; the first collecting basic information such as age, gender and course of study followed by questions investigating the exercise habits and sports participation of the subjects. Finally, participants were asked to complete the ORTO-15 questionnaire, which was used to evaluate orthorexia tendencies.

The ORTO-15 is a 15 item self-administered questionnaire designed to diagnose ON. It is one of only two diagnostic tools used to evaluate ON, and the most widely used [24]. Adapted from the 10 item Bratman test [25], the ORTO-15 contains 15 questions which are based on an individual’s attitudes on the choice, purchase, preparation and consumption of foods deemed healthy by the individual [24, 25]. Each question is answered using a Likert scale with 4 responses (always, often, sometimes, never), with a split of questions used to investigate the rational, cognitive and emotional areas of food choice [25, 26]. For each individual question, the different responses correspond to a number (1–4), with those answers being the most indicative of orthorexia given the lower numbers. The total points range from 15 to 60 and scores below 40 are considered to indicate ON tendencies according to the original authors of the test [25]. There has been some discussion as to whether 40 is too high and a lower value of 35 should be used, with many studies choosing to do so [27]. This study used the established cutoff of 40 [25].

The ORTO-15 was originally developed in Italian and has since been translated, adapted and validated into many different languages such as Turkish [28], Portuguese [29], Hungarian [30], Polish [24], German [31], and Spanish [26]. The English version used for this study was adapted from the original Italian by Donini et al. [25].

Some studies, such as a recent validation of the questionnaire in Poland suggested that the ORTO-15 has good repeatability and a satisfactory reliability (Cronach’s alpha 0.7–0.9) [32] and a recent review on orthorexia literature confirmed this, with a Cronbach alpha coefficient ranging from 0.83 to 0.91 [33]. Therefore, despite some criticism, it is acknowledged that ORTO-15 is the presently the only well accepted method of screening for symptoms of ON [33].

### Data analysis

The ORTO-15 scores and total hours of exercise were calculated for all participants. The data was analyzed using SPSS version 12.0 and the level of statistical significance was set to *P* < 0.05. Normality was assessed both the total hours of exercise and the ORTO-15 scores between the two groups were compared using two sample *t* tests. Further comparisons were made between the students undertaking ≤ 10 h of exercise per week to those completing 10 or more and student athletes competing in aesthetic and weight dependent sports vs. those competing in tactical and technical sports. Pearson’s product correlation coefficients were used to assess the relationship between the volume of exercise and ORTO-15 scores. Chi-square tests were used to test whether the different conditions (e.g., sports team vs. non-sports team) differed by gender. Data is presented as mean ± standard deviation (SD). To estimate the magnitude of the changes Cohen’s *d* effect sizes (ES) were calculated with the magnitude of effects considered either small (0.20–0.49), medium (0.50–0.79) and large (> 0.80).

## Results

The mean ORTO-15 score for all students was 36.9 ± 3.9. More females (*n* = 141) then males (*n* = 74) took part. Mean ORTO-15 scores for all males was 36.4 ± 4.3 and for all females 37.2 ± 3.8; these, and the mean scores for males playing sport (36.8 ± 4.0) vs. not-playing sport (37.1 ± 4.0) and females playing sport (36.8 ± 4.0) and not-playing sport (36.9 ± 3.9) were not significantly different (*P* > 0.05).

Although there was no statistical difference between the two groups of participants, both had a mean ORTO-15 score below the 40 cutoff, with a total of 76% of participants scoring less than 40. The number of females scoring below 40 was 75% and the number of males 78%.

There were 28 sports represented by participants who competed in sport which included a mixture of team, aesthetic and technical sports (Table 1). Because of the low number of respondents from some sports, an inter-sport comparison was not performed. However, a sub-analysis on scores for students who competed in aesthetic and weight dependent sports vs. those who competed in more tactical and technical sports or those less reliant on relative body weight did not reveal any significant differences in ORTO-15 scores (*P* = 0.414; Table 2).

**Table 1:** Average number of hours of exercise completed each week and average ORTO-15 score for individual sports

Sport	All (*N*)	Females (*N*)	Males (*N*)	Average hours (mean ± SD)	ORTO-15 score (mean ± SD)
< 400 m track sprinting^a^	2		2	14 ± 0.5	35.5 ± 0.5
Basketball	1		1	16	30
Cheerleading^a^	16	16		10.17 ± 4.64	37.75 ± 3.53
Cricket	1		1	14	35
Cycling^a^	1	1		2	42
Dance^a^	12	11	1	8.89 ± ± 3.1	37.08 ± 2.53
Diving^a^	1	1		14	34
Football	4		4	8.13 ± 5.2	35.35 ± 4.71
Golf	1		1	14	35
Gymnastics^a^	2	2		8.25 ± 1.25	39.5 ± 3.5
Hockey	3	2	1	9 ± 0.82	43.67 ± 3.4
Judo^a^	2	1	1	8	36.5
Korfball	1	1		10	39
Lacrosse	6		6	9.25 ± 2.12	35.67 ± 2.92
Netball	3	3		10 ± 4.55	37.67 ± 3.09
Olympic weightlifting^a^	1		1	15	37
Orienteering	1	1		6	37
Rowing^a^	2	1	1	22.75 ± 3.25	38 ± 5
Rugby	15	1	14	10.14 ± 3.35	34.47 ± 4.03
Sepak takraw	2		2	9.75	28
Swimming^a^	12	9	3	11.58 ± 4.35	36.33 ± 4.96
Taekwondo	1		1	11	33
Table tennis	1		1	18	37
Trampolining^a^	1	1		14	33
Triathlon^a^	1	1		16	37
Ultimate Frisbee	4	1	3	8.38 ± 3.97	39 ± 2.55
Volleyball	1		1	12	42
Water polo	32	22	10	9.64 ± 3.78	35.53 ± 3.68

^a^Categorised as aesthetic or weight dependent sports based on previously suggested classifications [13, 35, 36]

**Table 2:** Participant characteristics, exercise habits and ORTO-15 score from students who competed in aesthetic/weight dependent sports vs. technical and tactical/non-weight dependent sports

	Weight dependent sports	Non-weight dependent sports	Effect size	*P* value
Total number of participants (% of total)	48 (41%)	68 (59%)	–	–
Number of females (% of category)	40 (74%)	27 (40%)	–	0.0001*^b^
Number of males (% of category)	8 (16%)	41 (60%)	–	0.0001*^b^
Age (years)^a^	20 ± 2 (18–25)	21 ± 2 (18–27)	0.35	0.063
Weekly exercise (h)^a^	10.7 ± 4.0 (2.0–26.0)	9.5 ± 3.9 (2–20)	0.30	0.135
ORTO-15 score (15–60)^a^	37.1 ± 3.9 (30.0–45.0)	36.3 ± 3.9 (28.0–47.0)	0.20	0.303

*Represents significant difference

^a^Parenthesis represents minimum and maximum values. *n* = 116 in total

^b^Chi-square tests used for analysis

There was a difference between the mean hours of exercise between those who played sport and those who did not with those who competed in sport exercising an average of 10.0 ± 4.4 h a week and those who didn’t exercising an average of 3.6 ± 2.8 h per week (*P* = 0.0001). There were no differences in age or ORTO-15 scores between the student athletes and non-athletes (*P* > 0.05; Table 3); however, students completing ≥ 10 h of exercise per week were found to have lower ORTO-15 score then those performing ≤ 10 h/week (*P* = 0.008; Table 4). There was a significant negative correlation between the number of hours of exercise performed per week and ORTO-15 scores (*r* = − 0.213; *P* = 0.002).

**Table 3:** Exercise habits and ORTO-15 scores from students who compete in sports and those who do not

	Sports students	Non-sports students	Effect size	*P* value
Total number of participants (% of total)	116 (54%)	99 (46%)	–	–
Number of females (% of category)	67 (58%)	74 (75%)	–	0.0001*^b^
Number of males (% of category)	49 (42%)	25 (25%)	–	0.0001*^b^
Age (years)^a^	21 ± 1 (18–27)	21 ± 2 (18–25)	0.00	0.757
Weekly exercise (h)^a^	10.0 ± 4.3 (2.0–26.0)	3.6 ± 2.8 (0.0–15.0)	1.80	0.0001*
ORTO-15 score (15–60)^a^	36.6 ± 3.9 (28.0–47.0)	37.2 ± 3.9 (25.0–47.0)	0.15	0.279

*Represents significant difference

^a^Parenthesis represents minimum and maximum values. *n* = 215 in total

^b^Chi-square tests used for analysis

**Table 4:** Participant characteristics, exercise habits and ORTO-15 score from students who complete ≥ 10 vs. ≤ 10 h of exercise a week

	≥ 10 h	< 10 h	Effect size	*P* value
Total number of participants (% of total)	60 (28%)	155 (72%)	–	–
Number of females (% of category)	31 (52%)	110 (69%)	–	0.0001*^b^
Number of males (% of category)	29 (48%)	45 (31%)	–	0.0001*^b^
Age (years)^a^	21 ± 1 (18–27)	21 ± 2 (18–25)	0.00	0.897
Numbers competing in sport (% of category)	57 (95%)	59 (38%)	–	–
Weekly exercise (h)^a^	13.5 ± 3.3 (10.0–26.0)	4.6 ± 2.7 (0–9.5)	2.96	0.0001*
ORTO-15 score (15–60)^a^	35.6 ± 4.3 (25–47)	37.3 ± 3.6 (26–46.0)	0.43	0.008*

*Represents significant difference

^a^Parenthesis represents minimum and maximum values. *n* = 215 in total

^b^Chi-square tests used for analysis

It is important to note that for all analysis there was a gender imbalance between the different conditions analyzed (see Tables 2, 3, 4).

## Discussion

The main finding of this is that there were no differences in ORTO-15 scores between student athletes competing for a University sports team and non-student athletes not playing for a sports team, irrespective of gender and type of sport. However, ORTO-15 scores were lower in students undertaking ≥ 10 vs. ≤ 10 h of exercise per week, suggesting that volume of exercise might have a mediating influence on orthorexic tendencies. This finding was supported by the negative correlation between ORTO-15 scores and number of hours of exercise per week. This is the first study to examine ON in student athletes from Universities in the UK and provides new information on the prevalence of this newly described disorder in this population.

A previous study in Italian athletes found that compared with controls, the athletes scored significantly higher on the ORTO-15 [19]. This study provided the first indication that athletic populations might have a greater risk of ON than their less active counterparts. In contrast, in the present study, we did not find any differences in ORTO-15 scores between the student athletes and non-athlete controls. It was only when the groups were separated into those who completed > 10 h of exercise per week vs. <10 h/week that a small but significant difference in ORTO-15 scores emerged. Because Segura-García et al. [19] did not report the activity levels of the athletes in their study so it unclear if the discrepancy in finding between ours and their study is due to the fact the athletes in their study were performing more exercise then the student athletes in our study. Regardless, our findings suggest that student athletes who train more have higher ON symptoms. These findings are in agreement with a recent study, which found students reporting higher ON symptoms spent more time exercising [21]. While it was beyond the scope of this study to investigate the possible causes of ON in this population, previous studies have suggested that the greater pressure to perform, whether internally or externally, is known to be a risk factor for athletes developing an eating disorder [34]. Perhaps those who exercise ≥ 10 h/week are of a higher standard in their respective sports and, therefore, may be under greater pressure to perform well, especially if they are on some form of sporting scholarship, or on a team expected to do well within the University leagues. Our questionnaire did not capture the level of athlete but perhaps this is a consideration for future research.

Several studies suggest that eating disorder symptomology is higher in athletes who compete in aesthetic and weight dependent sports that focus on leanness and low body fat, such as gymnastics, swimming and cycling [13, 35, 36]. In the present study, we did not find any significant differences in ORTO-15 scores between the student athletes competing in these weight dependent sports and the student athletes who take part in technical and tactical sports less focused on leanness (e.g., rugby and hockey), suggesting there were no differences in ON symptomology in the different types of sports. Although these findings are preliminary, they suggest that the prevalence of ON symptoms might not be greater in weight dependent sports like often seen with clinical eating disorders such as anorexia nervosa [13, 15].

In this study, 76% of participants scored less than 40 on the ORTO-15 scale. Results similar to this were found by Dunn et al. [37] when examining a sample of US college participants, in which 71.2% scored below 40, as well as numerous other studies which have explored the frequency of ON symptoms amongst university and college students [32, 38–40]. This suggests that a high prevalence in those competing in sport is potentially masked by the high prevalence in students in general. On the other hand, there is limited data on other age groups such as middle-aged men and women, and therefore, it is hard to compare these findings to the general population [33] and conclude that students have a higher prevalence of ON.

The high frequency in which students score below the 40 cutoff on ORTO-15 questionnaire, both in this study, and in others [37] is well above that of any other recognized eating disorder such as Anorexia Nervosa and Bulimia Nervosa, both of which have an estimated prevalence of less than 2% in the general population [41]. One study [37], found a high frequency of participants scoring below 40 when using the ORTO-15 questionnaire. However, when further methods were used to assess eating behavior, they found that only 20% of their participants restricted a food type, and when asked whether participants believed their thoughts about food to impair their everyday activities, < 1% of the sample were deemed to have ON tendencies. This drastically lower prevalence is more similar to the frequency at which other established eating disorders are observed. Similarly, the original authors of the ORTO-15 used the questionnaire alongside the Minnesota multiphasic personality inventory, which gives an indicator of obsessive behavior, which suggests that the ORTO-15 as a sole measure is not advised for a clinical diagnosis [25].

There are many suggestions as to why the prevalence of ON and other eating or weight disorders might be higher in students. Some authors suggest that the transition to University and the associated increase in independence, stress, social pressure and low self-esteem can lead to dietary behavior change, especially in an effort to avoid the common weight gain, some students experience [42]. This change in dietary behavior may lead to the development of ON. Others suggest that students and young adults have many more influences to ‘healthy’ and ‘clean’ eating ideologies through the use of social media. Indeed, [3] found that young Instagram users were associated with higher orthorexic tendencies, with many users exposed to a high frequency of food and body images, which may encourage certain behaviors or thoughts [3]. However, with the widespread use of social media, this problem would be unlikely to be unique to students, but also other groups of individuals with a similar age and social media usage. Furthermore, eating disorders are caused by a complex interaction between a vast number of factors, making it difficult to determine exactly why students may have a higher prevalence [43].

This study has a number of limitations that need to be acknowledged. Among them is the questioned validity of the ORTO-15 questionnaire. Missbach et al. [31] concluded in their study that the ORTO-15 had some basic psychometric flaws, and even the creators of the test suggested that further investigation and additional questions should be considered due to the ORTO-15’s limits in discriminating some distinctive components of ON, such as obsessive traits [25]. Furthermore, it is evident across the literature that many researchers both alter the cut-off points (from < 40 to < 35), and the questions included when conducting their studies, especially in language translations, which leads to the validity and reliability being compromised due to a lack of standardization [33].

Another limitation with the ORTO-15 questionnaire is the tendency to possibly over estimate the number of people with ON symptoms, which has prompted a call for a new diagnostic tool [31]. The fact that several studies [19, 25, 37] all used additional measures to explore orthorexic tendencies suggests that the ORTO-15 alone might not be sensitive enough to make a distinction between those who have unusual, or even extreme healthy eating behaviors, from those who have behaviors that are pathological and impair daily life. We did not use additional measures, because, as this was only a preliminary study, we wanted to ensure the survey was as brief as possible for the participants; indeed, we felt that adding further questionnaires to the study would be off-putting for the students and reduce the number of participants who took part. In view of this, it is clear that is a vitally important a new screening and/or diagnostic tool for ON is developed.

A strength of the study was that it found similar results to numerous others which use the ORTO-15 questionnaire when it comes to the diagnosis of ON amongst student populations [32, 38–41]. This study had a large number of students (*n* = 215), who came from a range of different stages, courses, and exercise levels. This heterogeneous group is likely to be a good representation of the students in the UK. Finally, these findings are novel in that this also the first study attempting to delineate the prevalence of ON in student athletes in the UK.

There are several possible avenues for future work on ON symptoms in athlete populations. Foremost, it is important to explore the potential reasons as to why ON symptoms might increase with exercise volume in student athletes. It would also be useful to investigate ON symptoms in elite level athletes to assess the prevalence and motivations in this cohort and compare them to sub-elite populations, akin to the participants in this study. Studies should also extend upon our initial findings and investigate the prevalence of ON symptoms in weight dependent and non-weight dependent sports. Future studies may also look into the difference in ON prevalence between students and non-students to help give an insight into whether high frequencies are reported in other population groups. Having a better indication of this can help determine sensitivity of the ORTO-15, and whether it is currently too high to truly capture people with this eating disorder. Finally, future studies should include a higher number of participants, an equal balance of males and females, and include Universities in several different countries to assess potential inter-country differences in ON symptoms.

## Conclusion

In conclusion, this pilot study suggests that ON symptoms do not appear to be greater in student athletes than non-student athletes, or greater in those who take part in aesthetic and weight dependent sports. However, we did observe a small but significant increase in ON tendencies in individuals performing greater than 10 h of exercise per week. However, there is ongoing debate of the validity and use of the ORTO-15 questionnaire and this must be addressed in future studies if ON is going to become a recognized and diagnosable eating disorder. Studies with better screening tools for ON are required in athletic populations.
